# Magnesium Binding by *Cyberlindnera jadinii* Yeast in Media from Potato Wastewater and Glycerol

**DOI:** 10.3390/microorganisms11081923

**Published:** 2023-07-28

**Authors:** Anna M. Kot, Stanisław Błażejak, Klaudia Nosek, Alicja Synowiec, Anna Chlebowska-Śmigiel, Katarzyna Pobiega

**Affiliations:** Department of Food Biotechnology and Microbiology, Institute of Food Sciences, Warsaw University of Life Sciences, Nowoursynowska 159C, 02-776 Warsaw, Poland; stanislaw_blazejak@sggw.edu.pl (S.B.); klaudianosekk@gmail.com (K.N.); alicja_synowiec@sggw.edu.pl (A.S.); ach.smigiel@gmail.com (A.C.-Ś.); katarzyna_pobiega@sggw.edu.pl (K.P.)

**Keywords:** fodder yeasts, potato wastewater, glycerol, magnesium

## Abstract

The aim of this study was to determine the magnesium-binding capacity of *Cyberlindnera jadinii* yeast in media prepared from potato wastewater and glycerol (after biodiesel production), supplemented with magnesium salt. The research was carried out in two stages. In the first, the ability to binding magnesium by yeast in media supplemented with various doses of this element was tested. In the second stage, after selecting the appropriate dose of magnesium, the culture was carried out in a bioreactor. The composition of the yeast biomass was also analysed in terms of lipids and protein content and amino acid composition. Studies have shown that this type of medium can be used as a culture medium for the growth of *C. jadinii* yeast. In the first stage of the study, the most magnesium (8.97 mg/g_d.m._) was bound by yeast cells after 48 h of cultivation in a medium supplemented with the addition of magnesium at a dose of 2 g/L. In the second stage of the research, the highest magnesium content in the biomass (7.9 mg/g_d.m._) was noted after 24 h of cultivation in the same medium. The lipid and protein contents in the biomass obtained after 24 h of cultivation in the bioreactor were 6.35 and 43.73%, respectively. The main fatty acids present in the yeast lipids were oleic acid (59.4%) and linoleic acid (8.6%). Analysis of the amino acid profile of the proteins showed the highest proportions were glutamic acid (13.7%) and aspartic acid (11%).

## 1. Introduction

Magnesium is called the element of life. It plays an important role in the biochemical processes that take place in living organisms. It improves the functioning of metabolic processes and activates over 300 enzymes, which are responsible, among others, for the stabilisation of nucleic acids and ribosomes [1]. Magnesium is involved in the synthesis of macro-energy compounds, and also supports the maintenance of a proper mineral balance in the body [2]. Today, society struggles with a deficiency of this element, which is often associated with the initiation of pharmacological supplementation with agents that are widely available, but their absorption does not usually exceed 15% [3,4]. The bioavailability of magnesium can be improved by administering it in the form of bioplexes, that is, combinations of proteins with minerals. These combinations connect the value of the organic part, that is, the protein, with the micronutrient. They are characterised by better absorption in the digestive tract of humans and animals [5].

*Cyberlindnera jadinii* yeast has the ability to bioaccumulate various elements, including magnesium and selenium in amounts significantly exceeding the natural needs of the cells [6,7,8,9]. This yeast has been approved for consumption by the United States Food and Drug Administration (FDA) and are on the GRAS (Generally Recognized as Safe) list [10]. The possibility of using *C. jadinii* as a foodstuff was first described by German employees of the Für Gärungsgewerbe Institute in Berlin during World War I [11]. The biomass of these yeasts is characterised by a high protein content in the dry matter, which, depending on the cultivation conditions, ranges from 40 to 50%. These proteins are distinguished by above-average nutritional value and digestibility of about 50%. Moreover, the biomass of *C. jadinii* contains less nucleic acids compared to other yeast species. Usually, it is about 10% in the dry cell material; for comparison, the nucleic acid content in *Saccharomyces cerevisiae* cells is about 16%. In addition, the large dimensions of *C. jadinii* facilitate the separation of cells from the media after a specified culturing time [12]. The yeast binds magnesium in a two-step process; one of the stages is dependent on metabolism, while the other does not require energy expenditure. The first stage is quick (usually a few minutes), independent of temperature and metabolism, and requires no energy. The initial binding of magnesium involves the microbial cell wall, where the element is chemisorbed to functional groups with a negative charge of external mannoproteins located in the wall structure [13]. An important role in the process of binding elements is assigned to the hydroxyl, carboxyl, phosphate and amino groups that occur in the outer layer of the cell wall. The mannoprotein layer of the cell wall determines its permeability and provides a protective barrier. The second stage, called bioaccumulation, is longer, metabolically dependent and only occurs in living yeast cells. It consists of the active transport of metal ions through the structures of the cell wall to the cytosol [14]. Temperature, pH and number of cells, as well as the presence of other ions, have a significant impact on magnesium biosorption by yeast. The optimum temperature for this process is 25–35 °C and a pH of 4.0–8.0. An excessive number of yeast cells in the culture medium may inhibit the binding of metal ions [15]. The uptake of magnesium by yeast is carried out by transport proteins such as Alrlp and Alr2p, which are homologues of CorA (a term for cobalt tolerance proteins) found in yeast and other fungal cells [16]. These proteins are part of the MIT protein family, which are part of the cytoplasmic membrane, and their expression is related to the concentration of Mg^2+^. They show similarities to each other in terms of molecular weight, structure as well as isoelectric point. They have very similar molecular weights, and the structure is based on a single polypeptide chain. They operate on the principle of protein ion channels, generating a potential difference on both sides of the cell membrane. Other protein transporters, such as Lpe10, which belong to CorA homologues and are located on the inner mitochondrial membrane of yeast, also participate in the process of magnesium bioaccumulation [17].

*C. jadinii* yeast is a so-called wild yeast, which, compared to *Saccharomyces cerevisiae*, is characterised by low nutritional requirements in terms of digestible carbon and nitrogen sources, which, in turn, makes it possible to cultivate it from industrial waste. Many studies [18,19,20] showed that the process of producing *C. jadinii* yeast biomass can be carried out in media containing waste glycerol and deproteinized potato wastewater. Untreated glycerol waste generated during biodiesel production contains approx. 50–70% glycerol, as well as methanol, free fatty acids, water, monoacylglycerols, diacylglycerols, phospholipids, tocopherols, soaps, salts, dyes and catalyst residues [21,22]. Potato wastewater is not rich in carbon sources (sugar content 0.3–1.5%) and due to its high protein content (up to 1%), it can act as a nitrogen source in microbiological media [23]. The combination of these two types of waste makes it possible to obtain a higher yield of yeast biomass than when it is grown only in potato wastewater. As a result, it is possible to manage troublesome waste, with the simultaneous production of yeast biomass [24].

Bioplexes are characterized by better bioavailability. They are an alternative to pharmaceuticals. This makes it possible to supplement the diet of both humans and animals with microelements derived from organic compounds. Industrial waste can be a cheap and widely available source of culture media components. So far, research has been carried out on the possibility of binding magnesium by yeast, but the substrate prepared from potato juice water has not been used for this purpose. The aim of this study was to determine the natural magnesium-binding capacity of *C. jadinii* ATCC 9950 yeast in media prepared from potato wastewater with glycerol and supplemented with the addition of a magnesium salt.

## 2. Materials and Methods

### 2.1. Biological Material

The fodder yeast strain *Cyberlindnera jadinii* ATCC 9950 from the American Type Culture Collection was used in this research. The yeast was stored frozen (with the addition of glycerol) at −80 °C. The vial was thawed, transferred to YPD agar medium and incubated at 28 °C for 48 h.

### 2.2. Glycerol from Biodiesel Production

In the experimental media, technical glycerol from a factory producing biodiesel from rapeseed oil (PKN Orlen, Trzebinia, Poland) was used as a carbon source for the yeast. The technical glycerol contained 80% glycerol, 15% water and 0.3% methanol (data from PKN Orlen).

### 2.3. Deproteinized Potato Wastewater

The deproteinized potato wastewater was prepared in laboratory conditions according to methodology in [25] based on the stages of the potato starch production process used in industrial production factories. The potato wastewater contained 0.22 ± 0.02 g/100 mL nitrogen, expressed as NH_4_ (determined by the Kjeldahl method) and 0.66 ± 0.01 g/100 mL total sugars, expressed as glucose (determined by the Miller method).

### 2.4. Culture Media

The control medium was prepared from deproteinized potato wastewater and a glycerol fraction, which was added in such an amount that the final concentration of glycerol in the medium was 5% (*w*/*v*). The experimental media was enriched with MgSO_4_·7H_2_O salt (p.a., Avantor Performance Materials, Poland) so that the concentration of magnesium ions was 0.5, 1.0, 1.5 or 2 g/L. The initial pH of the media was adjusted to 5.0 ± 0.1 prior to the sterilisation process (121 °C/15 min, HICLAVEHG-80 autoclave, HMC Europe, Tuessling, Germany).

### 2.5. Inoculum Preparation

The yeast inoculum was prepared by inoculating 100 mL of YPD liquid medium (glucose 20 g/L, peptone 20 g/L, yeast extract 10 g/L; B.T.L., Łódź, Poland). Yeast colonies were taken with a loop from a Petri dish (cultivated at 28 °C for 48 h). The inoculum was grown in 500 mL flat-bottom flasks for 24 h at 28 °C on a reciprocating shaker (200 rpm, Sm-30 Control, Edmund Bühler GmbH, Bodelshausen, Germany). The number of yeast cells in the inoculum after this time was approximately 1 × 10^7^ CFU/mL (determined using a Thoma chamber).

### 2.6. Culture Conditions

First stage—Yeast cultures on a shaker. To inoculate the control and experimental media, 10 mL of the inoculum was centrifuged at 3500 rpm for 10 min (Centrifuge 5804 R, Eppendorf, Hamburg, Germany). The supernatant was removed, 10 mL of sterile water was added to the biomass and it was centrifuged again. After this, the supernatant was again removed and 10 mL of the appropriate medium (control or experimental) was added to the centrifuged biomass. The entire mixture was transferred to 500 mL flat-bottom flasks containing the culture medium (90 mL). The yeast were cultured for 96 h at 28 °C on a reciprocating shaker (200 rpm, Sm-30 Control, Edmund Bühler GmbH, Bodelshausen, Germany).

Second stage—Cultures in a bioreactor. Based on the results obtained during cultivation on the shaker, a dose of magnesium supplementation was selected, which was used during cultivation in a laboratory bioreactor (Bioflo 3000, New Brunswick Scientific, Hamburg, Germany). For this purpose, 3.5 L of medium was inoculated with the yeast inoculum (the inoculum volume relative to the medium was 5%). The culture was agitated vigorously (the agitator shaft rotation speed was 300 rpm) and oxygenated at an air flow of 2.0 *v*/*v*/*m*. The cultivation was carried out for 72 h at 28 °C.

### 2.7. Biomass Yield and Optical Density

The biomass yield was determined by the weight method. A 10 mL volume of the culture medium was collected and then centrifuged at 10,000 rpm for 10 min. The supernatant was removed and the biomass was suspended in 10 mL of sterile water and centrifuged again. After this process, the supernatant was discarded and the wet biomass was dried at 85 °C overnight. The sample was weighed and the cellular biomass yield in g_d.m._/L (grams of dry matter per litre of medium) was determined from the difference in mass.

In order to measure the optical density, 2 mL of culture medium was taken. The sample was centrifuged (5000 rpm for 5 min). After this period, the supernatant was discarded and 2 mL of distilled water was added to the biomass, mixed and centrifuged again. Then, the supernatant was discarded again and the wet biomass was resuspended in 2 mL of sterile water and the absorbance was measured at λ = 600 nm (relative to sterile distilled water).

### 2.8. Analysis of the Composition of the Culture Media

The glycerol content in the post-culture media was determined using a chemical method [25]. The nitrogen content was determined by the Kjeldahl method (Büchi Digestion Unit K-435, Büchi Distillation Unit K-355, Flawil, Switzerland) [26], and the sugar content was converted to glucose by the spectrophotometric method (λ = 550 nm) using 3,5-dinitrosalicylic acid (p.a., Sigma-Aldrich, Saint Louis, MO, USA) [27].

### 2.9. Determination of the Magnesium Content

A 20 mL volume of medium was taken and centrifuged at 10,000 rpm for 10 min. The biomass was washed twice with sterile demineralised water and dried at 85 °C for 24 h. The dried biomass was ground and then the appropriate amount was mineralised (Büchi Digestion Unit K-435) in a mixture of nitric and perchloric acid (p.a., Avantor Performance Materials, Gliwice, Poland). The magnesium content in the biomass was determined by atomic absorption spectrometry (AA-600, Shimadzu, Kyoto, Japan) at λ = 285.2 nm. The result was given as the magnesium content in one gram of yeast dry matter (mg Mg^2+^/g_d.m._).

### 2.10. The Protein Content in the Yeast Biomass

The protein content in the yeast cell biomass was determined by the Kjeldahl method. For this purpose, 200 mg of dried yeast cell biomass was weighed, mineralised in 98% sulphuric (VI) acid (Büchi Digestion Unit K-435), steam distilled (Büchi Distillation Unit K-355) and titrated with 0.1 M hydrochloric acid solution (p.a., Avantor Performance Materials, Gliwice, Poland). The total protein content was calculated using the conversion factor 6.25 and expressed as g/100 g_d.m._ [26].

### 2.11. The Amino Acid Content in the Yeast Protein

The content of amino acids in the yeast protein was determined by the Moore and Stein method [28]. The dried and ground yeast biomass was mineralised in 6 M HCl (p.a., Avantor Performance Materials, Gliwice, Poland) at 110 °C for 24 h under a nitrogen atmosphere. After this, the samples were cooled and the hydrochloric acid was removed by evaporation under reduced pressure. The remaining amino acids were dissolved in a pH 2.2 buffer. The samples for determination of the sulphur-containing amino acids were prepared differently. Formic acid (p.a., Avantor Performance Materials, Gliwice, Poland) was added to the dried and ground biomass and incubated for 16 h at 4 °C. After the addition of 1 M HCl (p.a., Avantor Performance Materials, Gliwice, Poland) to the samples prepared in this way, they were mineralised at 125 °C for 23 h. The content of selected amino acids (aspartic acid, threonine, serine, glutamic acid, proline, glycine, alanine, cysteine, valine, methionine, isoleucine, leucine, tyrosine, phenylalanine, histidine, lysine and arginine) was determined by ion exchange chromatography with post-column ninhydrin derivatisation using an automatic amino acid analyser (Agros AAA 500) according to the manufacturer’s standard protocol (Ingos, Prague, Czech Republic) [28,29,30,31].

### 2.12. The Lipids Content and Their Profile

The lipid content was determined using the Bligh and Dyer extraction method with modifications [32]. Briefly, the cell wall was disintegrated by adding 200 mg of dry biomass to 10 mL of 1 M HCl. The samples were incubated for 2 h at 60 °C. After cooling, 5 mL of chloroform and 10 mL of methanol were added, and the samples were vigorously shaken for 30 min. Then, 5 mL of chloroform and 5 mL of 20% NaCl were added and shaken again for 30 min. The samples were centrifuged (3500 rpm for 10 min), and the lower chloroform layer containing the lipids was collected and transferred to a new tube. Chloroform was evaporated under a nitrogen atmosphere, and the total lipid content was determined in g/100 g_d.m._. After evaporation of the solvent, 0.5 mL of a 2 M KOH solution in methanol was added to the samples which were then placed in a thermostat at 37 °C for about 12 h to esterify the fatty acids. The resulting esters were analysed using a gas chromatograph with a GC-FID flame ionization detector (TRACE TM 1300 Gas Chromatograph, Thermo Fisher Scientific, Waltham, MA, USA). An RTX-2330 capillary column (60 m 0.25 mm 0.2 μm, Restek, Bellefonte, PA, USA) was used. Nitrogen was used as the carrier gas, the flow was set at 1.6 mL/min. The temperature was increased at a rate of 3 °C/min starting from 50 °C (3 min) to 250 °C (5 min). The sample injection temperature was 230 °C with the FID detector operating at 260 °C. Fatty acid methyl esters were identified based on retention times of standards (GLC 461, Nu-Chek Prep, Inc., Elysian, MN, USA).

**Figure 1:**
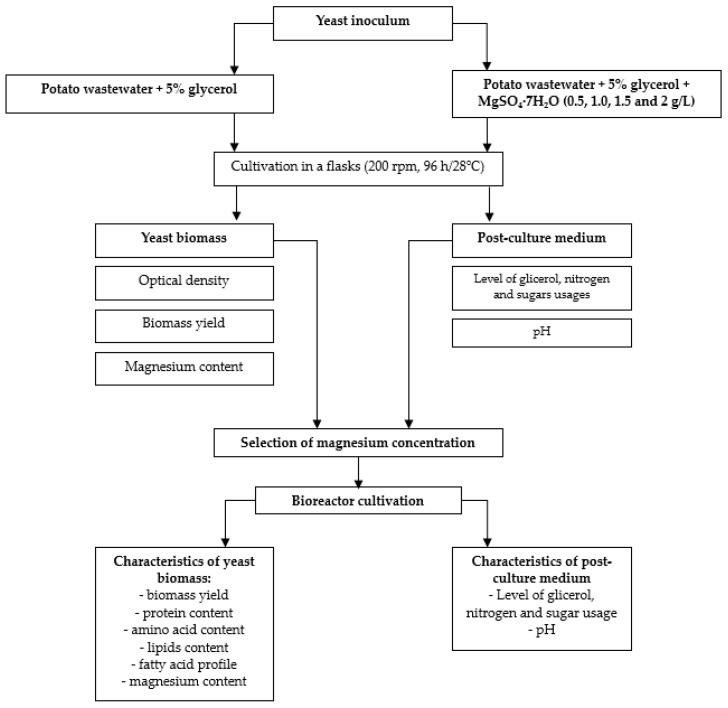
Experiment scheme.

The scheme of all performed experiments is shown in Figure 1.

### 2.13. Statistical Analysis of the Results

The results were statistically analysed using the Statistica 13.1 program. One-way analysis of variance (ANOVA) was performed. The normality of the residuals was checked with the Shapiro–Wilk test, and the homogeneity of variance with the Levene test. In order to determine homogeneous groups, a one-way analysis of variance was performed using Tukey’s test. A significance level of α = 0.05 was used.

## 3. Results and Discussion

Stage I—Yeast growth and utilization of culture media components

In the first stage of the work, the culturing of *C. jadinii* yeast was carried out in shaker flasks. On the basis of the first stage of the study, it was found that *C. jadinii* yeast was able to grow in both the control and experimental media supplemented with magnesium in concentrations of 0.5, 1.0, 1.5 and 2.0 g/L. The addition of magnesium did not significantly differentiate the obtained biomass yield in the studied concentration range (Table 1). The increase in the yield of cellular biomass lasted up to 96 h and after that time, the highest values of this index were achieved (35.3–36.1 g_d.m._/L). Studies describing the cultivation of *C. jadinii* ATCC 9950 yeast in various media are available in the literature. Kieliszek et al. [33] conducted a 48 h cultivation of yeast in YPD media supplemented with various doses of selenium. The mean value of the biomass yield in the YPD control medium after 48 h was 16.05 g_d.m._/L. A similar value (15.93 g_d.m._/L) of biomass yield after cultivation of *C. jadinii* ATCC 9950 in a YPD medium for the same time was also obtained by Kurcz et al. [19]. In this study, the biomass yield values after 48 h of cultivation in all media were within the range of 24.9–26.6 g_d.m._/L. This proves that potato wastewater supplemented with glycerol from biodiesel production can successfully replace the YPD medium in order to produce a large amount of yeast biomass, which is a natural magnesium biosorbent. This was confirmed by the results obtained during the analysis of the composition of the post-culture media. Regardless of the level of magnesium supplementation, the yeast used 97.6 to 99.2% of the initial content of glycerol introduced into the media during 96 h of cultivation. The presence of magnesium in the medium in the tested concentration range did not affect the process of nitrogen and sugar assimilation by the tested yeast strain from the culture medium, and the degree of use of these compounds was 58.3–65.3% and 71.8–73.8%, respectively (Table 2).

Stage I—Magnesium binding

**Figure 2:**
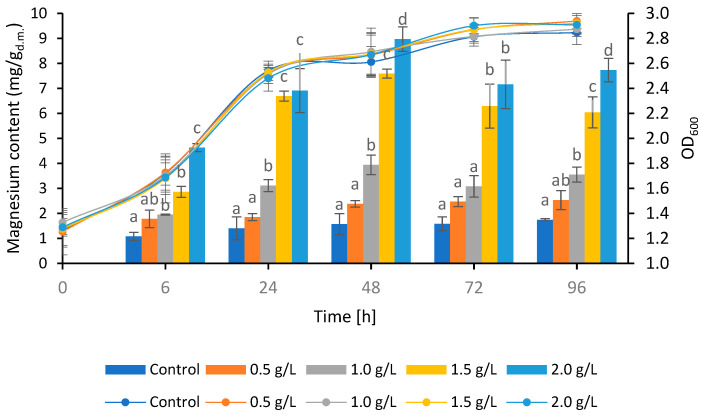
The amount of magnesium bound by yeast during cultivation in the control (0 g Mg^2+^/L) and experimental media (0.5, 1, 1.5 and 2 g Mg^2+^/L) (column chart) and changes in optical density during cultivation (line graph) ^a–d^—indicate non-significant differences between groups, determined using Tukey’s test, *p* < 0.05.

The addition of a magnesium salt to the experimental media resulted in an increase in the content of this element in the cell biomass compared to cultivation in the control medium without supplementation with Mg^2+^ cations. The highest amount of magnesium was bound to yeast cells (8.97 mg Mg^2+^/L) after 48 h of cultivation in the experimental medium with the addition of 2 g Mg^2+^/L (Figure 2). Błażejak et al. [34] found that a high concentration of Mg^2+^ ions at the end of the logarithmic growth phase can induce expression of the genes responsible for the intracellular accumulation of this element’s cations in *C. jadinii* ATCC 9950. It was also noted that cultivation for longer than two days resulted in a decrease in the magnesium content in the yeast biomass in the experimental media supplemented with 1.0, 1.5 and 2.0 g Mg^2+^/L. For the YPD control medium and a dose of 0.5 g Mg^2+^/L, no such dependency was observed. It can be assumed that the binding of the element to the yeast cell wall took place up to 48 h of cultivation, while the element was actively transported inside the cell as the cultivation time was extended [34]. This may mean that yeast in the logarithmic growth phase have a particularly high demand for magnesium. The rapid assimilation of magnesium in the logarithmic phase was due to the vitality of the yeast, which was intensively budding. At this stage of growth, an essential factor for proliferating cells is the presence of Mg^2+^ ions in the environment [35].

In another study by Błażejak et al. [36], the submerged cultivation of *C. jadinii* ATCC 9950 yeast was carried out in YPD medium with the addition of the magnesium salt MgSO_4_·7H_2_O at doses of 1.25 and 2.0 g Mg^2+^/L. The highest amount of magnesium (5.47 and 5.65 mg Mg^2+^/g_d.m._) was bound after 24 h of cultivation for media supplemented with magnesium ions in doses of 1.25 and 2.0 g Mg^2+^/L, respectively. The time required for the Mg^2+^ ion binding process was much shorter than that noted in these studies, and the values obtained were much lower, which suggests that the use of potato wastewater and glycerol allowed for prolongation of the logarithmic growth phase and, consequently, increased the magnesium content in the yeast biomass. The active acidity of the culture medium has a particular influence on the sorption process of magnesium ions by yeast and its subsequent accumulation in the form of bioplexes [37]. Before the start of the propagation process, the pH was adjusted to 5.0 in all media. A significant increase in pH was noted after the first day of cultivation, and the value of this indicator increased to 7.9–8.2 (Table 2) and remained at a similar level until the end of cultivation. The increase in pH was a consequence of the life processes of yeast and the production of secondary metabolites by yeast after the transformation of the nitrogen compounds present in the culture medium [38]. It cannot be ruled out that the increase in pH that took place during cultivation of the various yeast strains in the media containing potato wastewater as a nitrogen source [25,38,39] contributed to the increase in the amount of magnesium bound by *C. jadinii* ATCC 9950 yeast cells. Błażejak et al. [40], in order to investigate the influence of the pH of the culture medium on this process, used YPD media with an initial value of the active acidity equal to 5.0, 6.0 and 7.0, and supplemented with Mg^2+^ ions at a dose of 1.25 g Mg^2+^/L. A positive effect of a higher pH of the medium on the magnesium content in yeast cells was found. The highest content of magnesium (4.26 mg/g_d.m._) in the *C. jadinii* ATCC 9950 biomass was found in yeast after 48 h of cultivation in the medium at pH 7.0. This is because when the pH is neutral or slightly alkaline due to the lower concentration of hydrogen ions in the medium, lone pairs of yeast cell wall ligands more easily coordinate with metal ions than in an acidic environment. This increases the ability of yeast cells to bind metal cations from the culture medium.

Stage II—Yeast growth and utilization of culture media components

The second stage of the research consisted of the cultivation of yeast in a bioreactor, which was carried out in a medium supplemented with Mg^2+^ cations (in the form of MgSO_4_·7H_2_O) so that the concentration of this element was 2 g Mg^2+^/L. The choice of this concentration was justified by the highest amount of magnesium bound by the yeast biomass in the media supplemented with this element during cultivation on the shaker. The cultivation time in the bioreactor was 72 h.

*Cyberlindnera jadinii* yeast were able to grow intensively when grown in a bioreactor in a medium prepared from potato wastewater and glycerol with the addition of 2 g Mg^2+^/L. The biomass yield increased steadily during the 72 h of cultivation (Table 3). At the beginning of the cultivation, the mean value was 0.6 g/L, and after 24 h it increased to 19.3 g/L. The highest yield of biomass (50.6 g_d.m._/L) was noted after 72 h. By this time, the yeast had used 93.3% of the glycerol, 55.8% of the nitrogen compounds and 76.1% of the reducing sugars. As in the submerged cultivation on the shaker (Table 2), the pH value increased during the 72 h cultivation and reached a value of 8.2.

Stage II—Magnesium binding

The amount of magnesium bound by the yeast cell biomass after 24 h of cultivation was 7.9 mg/g_d.m._. This was the highest result achieved during the 72 h of cultivation in the bioreactor. It was found that time significantly influenced the binding of magnesium with the cellular biomass of *C. jadinii*. With the extension of the experiment time, the amount of magnesium bound with the cellular biomass decreased. The maximum value (7.89 mg/g_d.m._) was obtained faster than in the case of submerged cultures (48 h), while the result was slightly lower during cultivation on the shaker when the maximum was 8.97 mg/g_d.m._. According to Nowak et al. [41], the lower content of magnesium associated with yeast cells could be due to better mass transfer in the bioreactor between the cells and the environment. The authors studied the magnesium-binding capacity of *S. cerevisiae* yeast in YPD medium in a bioreactor. MgCl_2_·6H_2_O salt was added to the medium so that the concentration of the element was 1.25 g·dm^3^. The results obtained by the authors in bioreactor were lower than the test results obtained during stationary culture. The authors suggested that magnesium bound to biomass during cultivation in a bioreactor due to the intensity of mixing is more strongly bound to the dry substance and perhaps a greater amount of magnesium is transported inside the cell.

Stage II—Composition of yeast biomass

The protein content and its amino acid profile as well as the lipid content and the percentage of fatty acids were analysed in the cell biomass obtained during cultivation in the bioreactor. *C. jadinii* yeast are not oleaginous strains, so they are not capable of synthesising and accumulating lipids in an amount exceeding 20% of the dry cell substance. The lipid content in the biomass of the tested strain during cultivation in the bioreactor was constant over time and ranged from 6.26 to 6.90 (g/100 g_d.m._). As reported by Ratledge [42], the lipid content in *C. jadinii* yeast cells is in the range of 5–10%, regardless of the culture conditions. The lipids produced by the yeast were mainly characterised by a high content of oleic acid (C18:1), reaching over 55%. Other fatty acids found in significant amounts in the cells of the tested yeast strain included palmitic acid (C16:0, about 6%), margaric acid (C17:0, 8–9%), stearic acid (C18:0, 8–11%) and linoleic acid (C18:2, about 9%). On this basis, it was found that the microbial lipids contained in *C. jadinii* ATCC 9950 yeast were a good source of oleic acid which belongs to the group of monounsaturated fatty acids and of linoleic acid, which is a polyunsaturated acid belonging to the n-6 group.

The protein content in yeast cells and its amino acid profile are important due to the possibility of using the obtained biomass as an ingredient for farm animal feed. It was found that the protein content in the biomass was the highest at 24 h (43.73 g/100 g_d.m._) and decreased with time. Jach et al. [43] reported that the proportion of proteins in the yeast dry cell biomass may even reach 53%. The decrease in protein content during cultivation was most likely caused by the biosynthesis of other biomass components. The yeast likely synthesized more extracellular polysaccharides [18].

The cultivation time had no significant effect on the content of individual amino acids in the proteins synthesised by *C. jadinii* ATCC 9950 yeast (Table 4). Sixteen amino acids were found in the protein of the tested yeast strain. The highest percentages belonged to glutamic acid (approx. 14%) and aspartic acid (approx. 11%). The yeast protein was also rich in leucine (approx. 7%), alanine (approx. 6.5%) and lysine (approx. 6%). The proportion of cysteine, a nutritionally important sulphur-containing amino acid, was about 1.5%. No methionine was found.

The amino acid content determines the nutritional value of animal, plant and microbial proteins. The highest protein content in the biomass and the amount of magnesium associated with the yeast biomass were obtained after 24 h of cultivation. Therefore, the amino acid content of the yeast protein obtained after this cultivation time was compared with the Food and Agriculture Organization (FAO)/World Health Organization (WHO) standard (Table 5) [44].

The comparison of the amino acid composition showed a higher content of histidine, valine, isoleucine, leucine, lysine, phenylalanine + tyrosine and threonine in the yeast protein of the tested strain than in the FAO/WHO standard. Due to the absence of methionine, the sum of the sulphur-containing amino acids was lower than in the standard protein profile. The profile of the amino acids synthesised by *C. jadinii* yeast depends on the composition of the culture medium, and, above all, on the type of nitrogen source, which is described, among others, by Somda et al. [45]. The authors cultivated *C. utilis* FMJ12 yeast in a medium prepared using mango waste supplemented with various nitrogen sources. Very large differences in the content of amino acids were found depending on the nitrogen source used. For example, *C. utilis* FMJ12 yeast did not synthesise methionine in the peptone medium, while in the other three variants of the medium its content was 1.67 g/100 g protein (yeast extract), 0.48 g/100 g protein (ammonium sulphate) and 0.03 g/100 g protein (ammonium nitrate). For *C. jadinii* ATCC 9950 yeast grown in a medium with potato wastewater, glycerol and magnesium, the dominant amino acid was glutamic acid, the highest content of which was 16.57 g/100 g of protein, which the authors found after cultivation in a medium with yeast extract [45].

## 4. Conclusions

The conducted research showed the possibility of using potato wastewater and waste glycerol from the production of biodiesel as cheap components of the culture medium for producing the cell biomass of *C. jadinii* ATCC 9950 yeast. By supplementing the culture media with Mg^2+^ cations, valorisation of the cell biomass of the tested yeast strain was achieved, which can be successfully used as a potential source of well-absorbed magnesium bioplexes to treat a deficiency of this element in the diet of animals and humans. The cell biomass of *C. jadinii* yeast obtained with a 3.5 L-scale bioreactor with a medium of potato wastewater and glycerol enriched with the addition of magnesium at a dose of 2 g Mg^2+^/L was characterised by a high content of magnesium, a valuable profile of fatty acids (oleic, linolenic) and a high content of proteins with a favourable amino acid composition due to the significant proportion of some exogenous amino acids (especially lysine, valine, leucine and isoleucine).

## Figures and Tables

**Table 1:** Changes in the yield of *C. jadinii* yeast biomass (g_d.m._/L) during cultivation in the control (0 g/L) and experimental media (0.5, 1, 1.5, 2 g/L).

Time [h]	Control	0.5 g Mg^2+^/L	1.0 g Mg^2+^/L	1.5 g Mg^2+^/L	2.0 g Mg^2+^/L
0	0.99 ± 0.08	0.90 ± 0.04	0.88 ± 0.05	1.00 ± 0.03	0.88 ± 0.01
6	5.57 ± 0.45	5.04 ± 0.32	5.54 ± 0.59	5.86 ± 0.53	6.05 ± 0.06
24	13.51 ± 1.56	16.03 ± 1.52	16.25 ± 1.86	14.15 ± 3.59	15.02 ± 2.05
48	25.70 ± 3.49	24.91 ± 2.03	26.63 ± 0.87	25.65 ± 2.26	25.44 ± 1.87
72	33.83 ± 3.61	34.59 ± 2.03	35.71 ± 1.16	34.54 ± 3.40	33.70 ± 3.13
96	36.10 ± 4.78	35.83 ± 3.83	35.86 ± 2.07	36.04 ± 2.75	35.29 ± 2.75

No significant differences were found for results obtained for the same cultivation time (ANOVA test).

**Table 2:** The degree of utilisation of glycerol, nitrogen compounds and reducing sugars from the media after 96 h of cultivation and pH values of the medium after this time.

Type of Medium	Level of Glycerol Usage (%) *	Level of Nitrogen Usage (%) *	Level of Reducing Sugar Usage (%) *		pH of Medium				
				0 h *	6 h *	24 h *	48 h *	72 h	96 h
Control	98.4 ± 1.4	58.3 ± 8.3	72.6 ± 2.3	5.0 ± 0.1	5.68 ± 0.04	8.24 ± 0.35	7.96 ± 0.20	8.01 ± 0.12 ^a^	7.93 ± 0.10 ^a^
0.5 g Mg^2+^/L	97.5 ± 0.1	61.0 ± 4.8	71.8 ± 2.2	5.0 ± 0.1	5.54 ± 0.03	8.22 ± 0.37	7.81 ± 0.24	7.89 ± 0.03 ^ab^	7.80 ± 0.15 ^a^
1.0 g Mg^2+^/L	99.2 ± 0.4	64.8 ± 2.5	73.8 ± 1.8	5.0 ± 0.1	5.67 ± 0.03	8.16 ± 0.36	7.74 ± 0.10	7.59 ± 0.31 ^b^	7.62 ± 0.08 ^ab^
1.5 g Mg^2+^/L	99.1 ± 0.5	64.1 ± 2.1	72.6 ± 1.6	5.0 ± 0.1	5.51 ± 0.05	7.86 ± 0.10	7.72 ± 0.20	7.72 ± 0.13 ^ab^	7,52 ± 0.08 ^ab^
2.0 g Mg^2+^/L	99.2 ± 0.4	65.3 ± 6.8	72.9 ± 1.1	5.0 ± 0.1	5.95 ± 0.06	7.86 ± 0.14	7.58 ± 0.24	7.62 ± 0.21 ^b^	7.45 ± 0.09 ^b^

* No significant differences were found for results obtained for the same cultivation time (ANOVA test); ^a, b^—indicate non-significant differences between groups, determined using Tukey’s test, *p* < 0.05.

**Table 3:** The characteristics of growth, utilisation of nutrients from media and the degree of magnesium binding during cultivation of *C. jadinii* yeast in a bioreactor.

Time [h]	0 h	24 h	48 h	72 h
Biomass yield (g_d.m._/L)	0.61 ± 0.09 ^a^	19.31 ± 0.73 ^b^	30.23 ± 0.85 ^c^	50.58 ± 1.27 ^d^
Level of glycerol usage (%)	-	15.0 ± 1.5 ^a^	60.2 ± 3.6 ^b^	93.2 ± 1.8 ^c^
Level of nitrogen usage (%)	-	11.53 ± 2.4 ^a^	39.3 ± 1.5 ^b^	55.8 ± 2.1 ^c^
Level of reducing sugar usage (%)	-	56.1 ± 2.7 ^a^	70.7 ± 1.6 ^b^	76.1 ± 0.8 ^c^
Medium pH	5.0 ± 0.1 ^a^	7.12 ± 0.21 ^b^	7.68 ± 0.23 ^c^	8.21 ± 0.09 ^d^
Magnesium content in yeast biomass (mg/g_d.m._)	-	7.89 ± 0.24 ^c^	4.65 ± 0.38 ^b^	2.83 ± 0.64 ^a^

^a–d^—indicate non-significant differences between groups, determined using Tukey’s test, *p* < 0.05.

**Table 4:** Lipid and protein content in the cell biomass of *C. jadinii* yeast during cultivation in the bioreactor, and profiles of the amino acids and fatty acids.

Time [h]	24 h	48 h	72 h
Lipid content (g/100 g_d.m._)	6.35 ± 0.68 ^a^	6.26 ± 0.42 ^a^	6.90 ± 0.95 ^a^
Proportions of fatty acids (%):			
C16:0	6.61 ± 0.85 ^a^	5.80 ± 0.53 ^a^	6.24 ± 0.71 ^a^
C17:0	8.45 ± 0.96 ^ab^	7.16 ± 1.12 ^b^	8.94 ± 0.93 ^a^
C18:0	8.03 ± 0.87 ^b^	10.92 ± 1.63 ^a^	7.89 ± 0.72 ^b^
C18:1	59.38 ± 3.13 ^a^	55.77 ± 2.06 ^b^	56.46 ± 2.17 ^b^
C18:2	8.56 ± 1.03 ^a^	9.05 ± 0.95 ^a^	9.13 ± 0.47 ^a^
C20:0	0.86 ± 0.10 ^a^	0.40 ± 0.07 ^b^	0.90 ± 0.24 ^a^
C24:0	1.25 ± 0.25 ^b^	2.56 ± 0.64 ^a^	1.53 ± 0.30 ^b^
Protein content (g/100 g_d.m._)	43.73 ± 2.24 ^a^	33.75 ± 1.82 ^b^	26.94 ± 1.61 ^c^
Proportions of amino acids (%):			
Aspartic acid	10.98 ± 0.42 ^a^	10.84 ± 0.14 ^a^	10.67 ± 0.16 ^a^
Threonine	5.21 ± 0.09 ^a^	5.37 ± 0.04 ^a^	5.44 ± 0.06 ^a^
Serine	5.53 ± 0.13 ^a^	5.47 ± 0.10 ^a^	5.59 ± 0.12 ^a^
Glutamic acid	13.72 ± 0.41 ^a^	14.46 ± 0.41 ^a^	14.70 ± 0.09 ^a^
Proline	4.07 ± 0.11 ^a^	3.57 ± 0.04 ^b^	3.63 ± 0.27 ^b^
Glycine	4.83 ± 0.12 ^a^	4.70 ± 0.05 ^a^	4.85 ± 0.02 ^a^
Alanine	6.43 ± 0.23 ^a^	6.28 ± 0.07 ^a^	6.64 ± 0.01 ^a^
Valine	5.82 ± 0.12 ^a^	4.98 ± 0.14 ^b^	4.81 ± 0.02 ^b^
Isoleucine	5.22 ± 0.08 ^a^	5.16 ± 0.10 ^a^	5.02 ± 0.08 ^a^
Leucine	7.35 ± 0.21 ^a^	7.05 ± 0.18 ^ab^	6.76 ± 0.07 ^b^
Tyrosine	2.83 ± 0.08 ^a^	2.94 ± 0.32 ^a^	2.67 ± 0.22 ^a^
Phenylalanine	3.91 ± 0.05 ^a^	3.68 ± 0.20 ^a^	3.36 ± 0.11 ^b^
Histidine	2.69 ± 0.14 ^a^	2.59 ± 0.13 ^a^	2.53 ± 0.02 ^a^
Lysine	6.23 ± 0.20 ^a^	5.76 ± 0.10 ^b^	6.16 ± 0.03 ^a^
Arginine	5.17 ± 0.31 ^a^	4.29 ± 0.07 ^b^	3.30 ± 0.01 ^c^
Cysteine	1.64 ± 0.03 ^b^	1.47 ± 0.38 ^b^	1.81 ± 0.04 ^a^

^a–c^—indicate non-significant differences between groups, determined using Tukey’s test, *p* < 0.05.

**Table 5:** Comparison of the amino acid composition of the *C. jadinii* yeast protein with the FAO/WHO standard.

Amino Acid (g/100 g Protein)	Yeast Protein after 24 h of Cultivation in the Bioreactor	FAO/WHO Standard (2007)
Histidine	2.7	1.5
Valine	5.8	3.9
Isoleucine	5.2	3.0
Leucine	7.3	5.9
Lysine	6.2	4.5
Methionine + cysteine	1.6	2.2
Phenylalanine + tyrosine	6.7	3.8
Threonine	5.2	2.3

## Data Availability

The data presented in this study are available on request from the corresponding author.
